# DHA‐Rich Algae Feed Modulates Atlantic Salmon Health, Microbiota and Stress Response

**DOI:** 10.1155/anu/4185490

**Published:** 2026-07-02

**Authors:** Jonas Mueller, Marvin Suhr, Joachim Molkentin, Irene Lautenschläger, Jannick Ehlers, Anna Simon, Stéphanie C. Hornburg, Corinna Bang, Henrike Seibel, Carsten Schulz

**Affiliations:** ^1^ Institute of Animal Breeding and Husbandry, Department for Marine Aquaculture, Kiel University, Kiel, Germany, uni-kiel.de; ^2^ Fraunhofer Research Institution for Individualized and Cell-Based Medical Engineering IMTE, Aquaculture and Aquatic Resources, Büsum, Germany; ^3^ Institute of Animal Nutrition and Physiology, Kiel University, Kiel, Germany, uni-kiel.de; ^4^ Department of Safety and Quality of Milk and Fish Products, Max Rubner-Institute, Kiel, Germany, mri.bund.de; ^5^ Institute of Clinical Molecular Biology, Kiel University, Kiel, Germany, uni-kiel.de

**Keywords:** Atlantic salmon, fish health, functional feed, microbiome, RAS, Schizochytrium, stress

## Abstract

Reducing the amount of fishmeal and fish oil in salmon feed has resulted in a constant decline in the health‐promoting long‐chain polyunsaturated fatty acids (LC‐PUFAs) EPA and DHA available for the fish. As such, enriching feeds with essential fatty acids of nonfish origin becomes increasingly important. Here, we investigated whether diets supplemented with the DHA‐rich microalgae *Schizochytrium limacinum* can improve production performance and health of Atlantic salmon reared in a recirculating aquaculture system (RAS). Atlantic salmon post‐smolts (~126 g) held in triplicate tanks were fed diets enriched with *S. limacinum* at low (2%; SL2) or high (14%; SL14) inclusion or a control diet (CD). Following 8 weeks of feeding, the fish were exposed to peracetic acid (PAA), a commonly used disinfectant for RAS. Sampling of different health indicators alongside profiling the intestinal bacterial communities associated with the digesta and mucosa was used to evaluate the health‐promoting effects of the functional diets. Including *Schizochytrium* in the diet improved feed conversion, growth, and protein retention in Atlantic salmon, most notably in fish receiving the SL14 diet. Fatty acids in whole‐body and muscle samples reflected dietary levels, where DHA levels were particularly increased. 16S rRNA amplicon sequencing revealed a significant effect of the diet on beta‐diversity in digesta but not in mucosa. Relative abundance of specific genera was not affected by *Schizochytrium* inclusion, and most bacteria belonged to either *Firmicutes*, *Proteobacteria*, or *Actinobacteria*. Treating the fish with PAA induced an acute stress response, but the increase in plasma glucose and ion levels was diet dependent. Overall, our results highlight that including *Schizochytrium* in the diet can improve growth performance and modulate the response of Atlantic salmon to acute oxidative stress.

## 1. Introduction

Aquaculture continues to grow at the fastest pace of all food‐producing sectors. As a consequence, feed for farmed fish has changed considerably over the past decade, meeting both demand and sustainability concerns. Modern salmon diets are low in finite marine resources and dominated by plant‐based ingredients such as soy, wheat, and rapeseed [1]. This resulted in an overall decline of n3 long‐chain polyunsaturated fatty acids (LC‐PUFAs), including eicosapentaenoic acid (EPA; 20:5n3) and docosahexaenoic acid (DHA; 22:6n3) available for the fish [2]. Further reductions down to a total share of ~10% in the marine ingredients, fishmeal, and fish oil are needed to sustain Atlantic salmon farming until the end of the century [3]. These low levels of fishmeal and fish oil inclusion make it necessary to enrich feeds with long‐chain n3‐PUFA from nonfish sources in order to maintain the health‐promoting properties of aquafeeds.

The LC‐PUFAs EPA and DHA both play important roles in a variety of biological functions, such as maintaining the integrity of cell membranes, promoting immune function, and supporting growth in fish [4, 5]. Their requirement must be met largely through dietary intake as biosynthesis of EPA and DHA in Atlantic salmon is limited [6]. Recent work indicates that essential fatty acid requirements in Atlantic salmon can be met primarily by DHA [7]. While fish oil is currently still the main source of DHA in salmon feed, novel sources of DHA from microbial origin have been explored in the last decade. The marine thraustochytrid, *Schizochytrium* sp. (referred to here as microalgae) is a particularly rich source of DHA, containing up to 25% of its dry matter (DM) DHA [8]. It can be cultivated at a relatively low cost, making it an ideal DHA source for supplementing aquafeeds [9, 10].

The physiological demand for LC‐PUFA is not constant but depends on the fish’s life stage as well as the rearing environment and may be particularly high under stressful conditions [4, 11]. Both EPA and DHA can be involved in the mitigation of stress in fish [11–13]. Diets enriched with EPA and DHA counteracted heat‐ and selenium‐induced oxidative stress in Pangasius [14], and low n3 LC‐PUFA levels in diets fed to meagre resulted in increased plasma cortisol following stress [15]. Furthermore, [5] reported lower mortality of Atlantic salmon fed diets high in EPA and DHA following a natural cardiomyopathy syndrome outbreak. In mice, DHA‐phospholipids were shown to ameliorate chronic stress‐induced intestinal inflammation [16].

Although many studies have focused on the DHA requirements in various fish species, the potential of this fatty acid to mitigate stress in fish remains understudied. Farmed fish encounter a variety of stressors frequently during production, such as handling procedures, impaired water quality, or disease treatment. Land‐based recirculating aquaculture systems (RAS) particularly pose the risk of increased stress levels, as stocking densities are high and water quality and water treatment can induce stress responses and impair fish health [17]. Managing RAS often requires strict biosecurity measures to control the bacterial load in the system. Oxidative biocides have emerged as efficient eco‐friendly disinfectants for aquaculture, showing low effective concentrations and nontoxic degradation products [18]. However, treatment with strong oxidants such as peracetic acid (PAA) and hydrogen peroxide can induce stress responses in the treated fish. PAA was shown to elicit a systemic stress response in Atlantic salmon and rainbow trout, inducing expression of key antioxidant genes alongside an increase in cortisol and glucose levels [19–21]. It remains unclear to what extent these potential effects could be mitigated by means of a functional diet.

In addition to the direct effects DHA can have on fish health, a DHA‐enriched diet may indirectly influence host health by modulating the intestinal microbiome. Diet composition is a key driver shaping the intestinal microbiota in fish [22], which is involved in many processes beyond digestion, such as pathogen protection and immune responses. It has been found in several mammalian studies that the polyunsaturated fatty acid DHA can significantly influence the intestinal microbiota. For example, feeding socially isolated mice a diet enriched with DHA provoked a shift in the intestinal microbiota composition [23], and supplementing DHA restored intestinal microbiota composition after intestinal dysbiosis was induced by feeding a high‐fat diet [24]. In fish, [25] found reduced alpha‐diversity in the digesta of salmon fed with a diet high in lipid n3 LC‐PUFA. Furthermore, the dietary content of PUFAs influenced the intestinal microbiota in golden pompano [26] and gilthead sea bream [27]. Although physiological effects of DHA in fish are an important area of research relating to aquaculture, studies investigating the effects of increased DHA levels in feed on the intestinal microbiota are currently lacking.

We hypothesized that a diet low in the marine ingredients fishmeal and fish oil but enriched with the microalgae *Schizochytrium limacinum* as a source of DHA would improve growth performance, modulate the intestinal microbiota composition, and enhance the resilience to stress in Atlantic salmon. In our previous study [28], we did not detect any negative effects when *Schizochytrium* was included in the diet at 8% inclusion. In this study, we specifically tested whether a high (14%) or low (2%) inclusion rate of *S. limacinum* had an effect on Atlantic salmon.

## 2. Material and Methods

### 2.1. Ethics Statement

All experimental procedures complied with the EU Directive 2010/63/EU on the protection of animals used for scientific purposes and the national animal welfare regulations (TierSchVersV) and were conducted in accordance with the ARRIVE guidelines. The experiment was approved by the Ministry of Agriculture, Rural Areas, European Affairs, and Consumer Protection (MLLEV, Kiel, Germany; V 244 – 86776/2021).

### 2.2. Feed Formulation

The spray‐dried *S. limacinum* biomass consisted of 42.0% lipid, 25.9% protein, 26.2 MJ/kg energy, and contained 21.4% DHA. Given that we used an 8% inclusion of *S. limacinum* in the diet in our previous study [28], we aimed at investigating a higher and lower inclusion rate in this study. Therefore, we designed three experimental diets for Atlantic salmon [29] on a DM basis: a control diet (CD) without microalgae, a diet with low (2%) *S. limacinum* (SL2), and a diet with high (14%) *S. limacinum* (SL14) inclusion. The diets were formulated to substitute different plant‐based constituents, as shown in Table 1, and reflected current trends in the reduction of marine ingredients in Atlantic salmon diets [1]. The experimental diets were isonitrogenous, isolipidic, and isoenergetic (Table 2). The feed was pelletized into 4 mm diameter pellets (Type 14U175, Amandus Kahl, Hamburg, Germany) at temperatures below 60°C, sieved, and stored at 4°C prior to and during the trial.

**Table 1:** Feed formulation of experimental diets in g/100 g dry matter (DM).

Ingredients (g/100 g DM)	CD	SL2	SL14
Fish meal^1^	15	15	15
*Schizochytrium limacinum*	0	2	14
Blood meal^2^	6	6	6
Gelatin^3^	5	5	5
Pea protein isolate^4^	6	6	6
Soy protein concentrate^5^	18	18	12.5
Wheat gluten^6^	11.8	11.1	11.3
Wheat starch^6^	15	14.5	13
Canola oil^7^	7	7	4.5
Fish oil^1^	5	5	5
Palm oil^7^	7	6.4	4.2
Biolysine^8^	0.6	0.6	0.6
Methionine^8^	0.15	0.15	0.15
Vitamin and mineral premix^4^	0.5	0.5	0.5
CaHPO_4_^9^	2	2	2
Cellulose^10^	1	0.8	0.3

^1^Bioceval GmBH & Co. KG, Cuxhaven; Germany;

^2^Saria SE & Co. KG, Selm, Germany;

^3^Gustav Ehlert GmbH & Co. KG, Verl, Germany;

^4^Emsland‐Aller Aqua GmbH, Golßen, Germany;

^5^EURODUNA Rohstoffe GmbH, Barmstedt, Germany,

^6^Kröner‐Stärke GmbH, Ibbenbüren, Germany;

^7^Cargill GmbH, Riesa, Germany;

^8^Evonik Industries AG, Esssen, Germany,

^9^Lehmann & Voss & Co. KG, Hamburg, Germany

^10^Mikro‐Technik GmbH & Co. KG, Bürgstadt am Main, Germany.

**Table 2:** Macronutrient and fatty acid composition (in % fatty acids) of the experimental diets is given as the mean of two replicate analyses.

**Proximate composition (g/100 g DM)**	**CD**	**SL2**	**SL14**

Dry matter	92.3	91.8	90.1
Crude protein	49.8	49.9	50.1
Lipid	22.4	22.4	22.6
Ash	6.4	6.7	7.4
Crude energy (MJ/kg)	24.2	24.2	24.2

**Fatty acid composition (g/100 g FA)**			

C14:0	2.56	2.52	2.55
C15:0	0.17	0.17	0.17
C16:0	31.17	29.61	26.26
C17:0	0.16	0.17	0.13
C18:0	2.95	2.84	2.30
C20:0	0.39	0.39	0.33
C22:0	0.16	0.17	0.15
C24:0	0.11	0.11	0.11
Total SFA	37.69	35.98	32.00
**Fatty acid composition (g/100 g FA)**	**CD**	**SL2**	**SL14**

C16:1n7	1.40	1.39	1.46
C18:1n9	28.90	28.41	19.56
C18:1n7	1.65	1.64	1.37
C18:1n5	0.10	0.10	0.10
C20:1n9	3.77	3.73	3.63
C22:1n11	6.05	6.05	6.30
C22:1n9	0.39	0.40	0.36
C24:1n9	0.30	0.30	0.29
Total MUFA	42.95	42.43	33.47
C18:2n6	11.20	11.03	8.58
C20:2n6	0.11	0.10	0.09
C20:4n6	0.09	0.10	0.14
C22:5n6	0.09	0.46	2.87
Total n6‐PUFA	11.53	11.73	11.78
C16:3n3	n.d.	n.d.	0.11
C18:3n3‐ALA	2.81	2.80	2.13
C18:4n3‐SDA	0.77	0.77	0.84
C20:4n3	0.16	0.18	0.34
C20:5n3‐EPA	1.71	1.74	1.99
C22:5n3	0.21	0.23	0.27
C22:6n3‐DHA	2.19	4.15	17.07
Total n3‐PUFA	7.83	9.87	22.75
C16:2n4	0.14	0.14	0.14
C16:3n4	0.28	0.28	0.28
Total PUFA	19.78	22.01	34.95
n3‐HUFA	4.26	6.30	19.67

Abbreviations: ALA, α‐Linolenic acid; DHA, docosahexaenoic acid; EPA, eicosapentaenoic acid; HUFA, highly unsaturated fatty acids with 20 or more carbon atoms and three or more double bonds; MUFA, monounsaturated fatty acids; PUFA, polyunsaturated fatty acids; SDA, stearidonic acid; SFA, saturated fatty acids.

### 2.3. Experimental Design

The general experimental design and setup followed those of a previously published study [30]. In brief, Atlantic salmon post‐smolts were obtained from Danish Salmon A/S (Hirtshals, Denmark) and acclimated for 1 month in a RAS (24 m^3^, turnover rate 4 × h^−1^), which had a drum filter (mesh size 40 µm, type KTS 8–12, Kunststoff Spranger, Plauen, Germany), a moving bed biofilter (Kunststoff Spranger, Plauen, Germany), a protein skimmer (FLOTOR, Kunststoff Spranger, Plauen, Germany), and a UV disinfection system (Aqua Medic, Cologne, Germany) for water treatment. The fish received a commercial salmon diet (Aller Aqua, Denmark) twice a day during the acclimation period. Atlantic salmon (~126 g) were randomly divided into groups of 20 individuals and stocked in nine tanks (150 L) of the RAS. Triplicate tanks were hand‐fed with the respective experimental diet (Table 1) twice daily (9:00 am and 2:00 pm) at 1% of the fish’s body weight. Throughout the experiment, water quality remained at levels suitable for Atlantic salmon (20.9 ± 2.1 psu, 15.1 ± 0.4°C, 7.4 ± 0.2 pH, 10.6 ± 0.2 mg/L O2, 0.07 ± 0.09 mg N/L NH_4_^+^, and 0.05 ± 0.02 mg N/L NO_2_^−^). The experiment lasted for 56 feeding days, and daily feed rations were adjusted twice (in weeks two and five of the experiment). At the end of the feeding trial, all groups were subjected to acute oxidative stress induced by treatment with PAA (WOFA‐steril classic, Kesla, Germany) at 2.5 µl/L water, which is commonly used as a prophylactic treatment in RAS [18, 31]. It is a stabilized PAA solution containing 40% PAA and 12% H_2_O_2_. For this purpose, the tank water inlet was closed, while continuous aeration in the tank ensured that oxygen levels were maintained above 85% saturation. Then, 375 µl of PAA solution was added to each tank, and the water inlet was opened again after 1 h.

### 2.4. Fish Sampling

**Figure 1:**
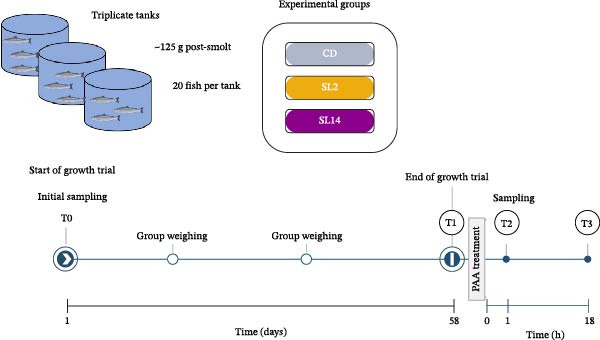
Experimental design of feeding trial. Atlantic salmon post‐smolts were fed for 8 weeks diets enriched with *Schizochytrium limacinum* at low (2%; SL2) or high (14%; SL14) inclusion or a control diet (CD). Subsequently, all groups were exposed to peracetic acid (PAA) at 2.5 ppt. The Atlantic salmon image was generated using Biorender.

Sampling was performed at the beginning (T0) and end of the feeding trial (T1), as well as 1 h (T2) and 18 h (T3) after exposure to the oxidative stressor (Figure 1). During the samplings, four fish per tank (*n* = 12 per treatment and *n* = 12 in total at T0) were quickly netted from the tanks and immediately killed by an overdose of buffered MS‐222 (0.3 mg/L).

The fish were measured for total length and weight, and a 2 mL blood sample was withdrawn from the caudal vein by puncture with a heparinized syringe (S‐Monovette, Sarstedt, Nümbrecht, Germany). The collected blood was centrifuged at 4000 × g for 8 min at 4°C, and aliquots of the resulting plasma were immediately frozen on dry ice and stored at −80°C for later analysis. During the samplings T0, T1, and T3, the fish were laterally opened and had their organs removed. Organ‐specific indices were determined by weighing both the liver and spleen.

At T1, the intestine was removed from the abdominal cavity, and digesta from the distal intestine were collected for microbiota analysis into 2 mL sterile collection tubes and flash‐frozen on dry ice. The intestine was cleaned by flushing it with 5 mL of sterile PBS. Thereafter, the intestine was longitudinally opened, and mucus was collected from the distal intestine with a sterile spatula. All samples were frozen on dry ice and subsequently stored at −80°C until DNA extraction.

At both T0 and T1, four fish per tank were taken and pooled for the analysis of whole‐body proximate composition. The fillets from nine fish per treatment (three per tank) were deskinned, homogenized (Grindomix GM200, Retsch GmbH, Haan, Germany), and stored at −40°C until analysis of fatty acids.

### 2.5. Proximate Composition of Algae, Diets, Whole Body, and Muscle

Approximately 50 g of each diet was homogenized with a mortar and pestle. Whole body samples were freeze‐dried (Alpha 1‐2 LD plus and Alpha 1‐4 LSC, Martin Christ Gefriertrocknungsanlagen GmbH, Osterode am Harz, Germany) and homogenized using a knife mill (GM 200, Retsch GmbH). Analysis of nutrients and gross energy was conducted following EU guideline (EC) 152/2009. Samples were dried at 103°C in a drying oven for 4 (12 h at 105°C in the case of muscle samples; ED 53, Binder GmbH, Tuttlingen, Germany), and DM was determined gravimetrically. The content was determined after combusting a subsample at 550°C (P300, Nabertherm, Lilienthal, Germany). Crude protein content was determined by the Kjeldahl method (InKjel 1225 M, WD30, Behr, Düsseldorf, Germany). A bomb calorimeter (C 200, IKA, Staufen, Germany) was used to determine the gross energy content. Crude lipids were extracted with petroleum ether from diet and whole‐body samples in a Soxhlet extraction system (Soxtherm, Hydrotherm, Gerhardt Königswinter, Germany) and subsequently quantified gravimetrically. Lipids from muscle tissue were extracted according to Smedes [32] using cyclohexane and 2‐propanol (VWR, Darmstadt, Germany) with modifications by Karl et al. [33].

### 2.6. Analysis of Fatty Acid Composition

Fatty acid analysis of diets, whole body, and muscle samples was conducted as described in Mueller et al. [28]. Briefly, a subsample of 20 mg dried algae and diet sample (weighed to the nearest 0.1 mg) was subjected to direct transesterification according to Griffiths et al. [34], but with 3 M methanolic HCl (Sigma–Aldrich, Taufkirchen, Germany) instead of boron trifluoride**-**methanol. Duplicate preparations were used for gas chromatography (GC) analysis. Lipids extracted from whole body and muscle without final drying [32] were transesterified into FAME using methanolic potassium hydroxide and subsequently used for duplicate GC analysis. Analysis of fatty acids was performed using a 7890A gas chromatograph (Agilent Technologies, Santa Clara, CA, USA) equipped with a 7683B autosampler, a split injection port (injection volume 1 µL, split 1:100), flame ionization detection, and a 100 m x 0.25 mm i.d. x 0.20 µm ‐ CP‐Sil 88 column (Agilent Technologies). Hydrogen with a constant flow of 1.6 mL min^−1^ was used as the carrier gas. Individual FAME were identified by comparison to known standards (SupelcoTM 37 Component FAME mix, PUFA Number 1, PUFA Number 3; all obtained from Sigma–Aldrich) in the range from C14:0 to C24:1n9. Fatty acid contents were calculated as mg of FAME/100 g of DM for algae and diet samples (Table 2) and as weight percentage (g FA/100 g FA) for whole‐body and muscle samples of duplicate analyses.

### 2.7. Microbiota Analysis

DNA from digesta was extracted using the QIAmp Fast DNA Stool Mini Kit (Qiagen, Germany, Cat. Number 51604), while mucosa samples were processed using the QIAmp DNA Microbiome Kit (Qiagen, Germany, Cat. Number 51704). Approximately 200 mg of digesta or mucosa was used as input. Bacterial cell walls of the respective samples were disrupted in a bead mill homogenizer (Fisherbrand, Thermo Fisher Scientific, Waltham, Massachusetts, US) using two cycles of 45 s each. Additionally, samples were incubated at 95°C for 5 min, centrifuged for 3 min at 20,000 rcf, and the supernatant was used for DNA extraction following the manufacturer’s instructions. Mucosa samples were extracted according to the manufacturer’s instructions without manual preprocessing. Preparation blanks were included in the extraction process as a quality control. Prior to sequencing, the 16S rRNA V3–V4 gene region (341F “CCTACGGGAGGCAGCAG” and 805R “GGACTACHVGGGTWTCTAAT” [35] was amplified by PCR in all samples. Multiplexed samples were sequenced on an Illumina MiSeq v3 platform at 2 × 300 bp. A detailed description of PCR cycling conditions and sequencing can be found elsewhere [36, 37].

Bioinformatic analysis of 16S rRNA gene sequences was performed using Quantitative Insights Into Microbial Ecology 2 (Qiime2, v.2021.2.0; [38]). Cutadapt was employed to remove leftover primers and spacers. Subsequently, filtering for low‐quality reads and chimeras and merging paired‐end reads were conducted using DADA2 [39] with truncation parameters of 277 and 221 for forward and reverse reads, respectively, and a truncation quality cutoff of two. ASVs occurring in at least two samples with a frequency of >25 were retained for taxonomic determination based on the SILVA reference database version 138.1 [40]. Only ASVs of bacterial origin and identified at the phylum level were kept for further downstream analysis.

### 2.8. Plasma/Liver Metabolites and Enzyme Activities

Plasma ions (Na^+^, Cl^-^) and glucose were determined using commercial dry chem slides on a Fuji Dry Chem NX500i (Fujifilm, Tokyo, Japan). Free cortisol in plasma samples was quantified using a commercial enzyme‐linked immunosorbent assay (ELISA) kit (Demeditec Diagnostics GmbH, Kiel, Germany).

### 2.9. Statistical Analysis

Statistical analysis and visualization were conducted in *R* (Version 4.3.1; R Core Team 2021, Vienna, Austria) with the *R* studio interface. First, an appropriate statistical model was defined, and second, model assumptions were checked by graphical residual analysis. Upon this, a Pseudo *R* ^2^ was calculated [41], and an analysis of variance (ANOVA) was conducted. For all response variables only available on a tank level (the production parameters and whole‐body composition of nutrients and fatty acids), models based on generalized least squares (gls; [42]) were used to evaluate the effect of the diet. When several observations were present for one replicate tank, a mixed effect model (lme; [43]) with a fixed effect of diet and a random tank effect was used. Lastly, in case a response variable was measured at more than one timepoint (before and after stress treatment), the mixed effect model included diet, timepoint, and their interaction as fixed effects and tank as a random effect. The residuals were assumed to be approximately normally distributed and homoscedastic. Multiple contrast tests [44] were conducted to compare across the factor diet based on Tukey. Multiple contrasts were chosen as the study aim was to compare the three dietary groups among each other and not to establish a dose–response curve. In the case of lme models, multiple contrasts were done by comparing least square means under lsmeans [45] and correcting for multiple comparisons based on Tukey.

To test for differences in beta‐diversity, permutational multivariate analysis of variance (PERMANOVA) with the Bray–Curtis distance as input was performed using the *adonis2* function from the *vegan* package [46]. Pairwise comparisons were conducted employing pairwise PERMANOVA. In addition, multivariate homogeneity of group dispersion was tested with factor diet using the *betadisper* function from *vegan*. To explore potential correlations among individual health parameters with the relative abundance of individual bacterial genera, a Pearson correlation coefficient was calculated for parameters and bacterial genera not influenced by the factor diet. For each health parameter, *p*‐values resulting from correlations across all bacterial genera were adjusted for multiple testing using the Benjamini–Hochberg procedure to control the false discovery rate (FDR). The FDR adjustment was applied separately for each health parameter.

## 3. Results

### 3.1. Fish Performance

Following restricted feeding over 56 days, fish growth measured as SGR and TGC was significantly affected by the diet (*F*(2,6) = 31.36 */* 27.71, *p*  < 0.001, respectively; Tables 3 and S1), while actual feed intake was not different among groups (*p*  > 0.05). SGR was lowest in CD and significantly improved in fish fed SL14 compared to both CD (*p*  < 0.001) and SL2 (*p* = 0.004). This effect was caused by a significantly lower feed conversion efficiency (*F*(*2*,*6*) *= 22.54*, *p* = 0.002) in salmon receiving SL14 compared to CD (*p* = 0.001) and SL2 (*p* = 0.01). Furthermore, fish fed SL2 and SL14 retained the ingested protein more efficiently (*p* = 0.035 & *p*  < 0.001 respectively) compared to CD, and PER was significantly improved in SL14 compared to CD (*p*  < 0.001) and SL2 (*p* = 0.004). None of the investigated organosomatic indices were affected by the diet, and no mortalities occurred during the experiment (Table 3).

**Table 3:** Growth performance and organ indices of Atlantic salmon fed experimental diets for 8 weeks.

Parameter	CD	SL2	SL14	ANOVA
IBW (g)	125.5 ± 2.1	127.8 ± 1.8	126.9 ± 1.8	0.687
FBW (g)	199.7 ± 2.4^a^	209.1 ± 3.4^ab^	219.1 ± 3.4^b^	0.013
SGR	0.83 ± 0.01^a^	0.88 ± 0.01^ab^	0.98 ± 0.02^c^	<0.001
TGC	0.58 ± 0.01^a^	0.64 ± 0.01^ab^	0.73 ± 0.02^c^	<0.001
FCR	1.24 ± 0.03^a^	1.18 ± 0.01^ab^	1.06 ± 0.02^c^	0.002
PER	1.75 ± 0.04^a^	1.86 ± 0.02^ab^	2.08 ± 0.04^c^	<0.001
PRE	32.52 ± 0.47^a^	34.81 ± 0.12^b^	38.73 ± 0.69^c^	<0.001
CF	0.93 ± 0.02	0.93 ± 0.02	0.98 ± 0.02	0.161
HSI (%)	1.30 ± 0.04	1.31 ± 0.05	1.38 ± 0.08	0.558
SSI (%)	0.10 ± 0.01	0.10 ± 0.01	0.10 ± 0.01	0.857
Survival (%)	100 ± 0	100 ± 0	100 ± 0	—

*Note:* Data is presented as mean ± SEM, with *n* = 3 tanks per treatment for performance parameters and *n* = 12 individuals for organ‐specific indices. A significant difference (*p*  < 0.05) among diet groups was assessed by Tukey’s multiple comparisons, and values not sharing common letters are significantly different. IBW (initial body weight); FBW (final body weight); SGR (specific growth rate) = (ln (FBW) − ln (IBW))/experimental days × 100; TGC = (weightfinal^1/3^ − weightinitial^1/3^) × 1000 / sum (water temperatures per feeding day); FCR (feed conversion ratio) = total feed intake (g)/weight gain (g); PER (protein efficiency ratio) = weight gain (g)/crude protein intake (g); PRE (protein retention efficiency) = crude protein gained (g)/crude protein intake (g) × 100; CF (Fulton’s condition factor) = weight/fish length^−3^ × 100; HSI (hepatosomatic index) = liver weight (g)/fish weight × 100; SSI (spleen‐somatic index) = spleen weight (g)/fish weight (g) × 100.

### 3.2. Proximate and Fatty Acid Composition

Proximate whole‐body composition was not significantly influenced by the diet, but the lipid content increased slightly with SL inclusion (Table 4). Overall, fatty acids in whole‐body and muscle samples reflected dietary levels (Tables 4, 5), although several deviations were present. The saturated fatty acid palmitic acid (C16:0) increased in the muscle and whole‐body samples with SL inclusion, while dietary levels decreased with the increasing SL inclusion rate. In contrast, stearic acid (C18:0) contents decreased with SL inclusion in diets (Table 2), muscles (Table 5), and whole‐body samples (Table 4). Oleic acid (C18:1) in muscle and whole‐body samples reflected the decreasing levels in the diet when SL was included (Table 2). The concentration of dihomo‐γ‐linolenic acid (DGLA, C20:3n6) was reduced in muscle and whole‐body samples of fish fed the SL diets. Docosahexaenoic acid (DHA; C22:6n3) and Docosapentaenoic acid (DPA; C22:5n3) were significantly enriched in whole‐body and muscle samples, mirroring the increased concentrations of both fatty acids in the diet. Furthermore, EPA concentrations in the whole body were increased in fish fed SL14, reflecting the slightly increased levels in the diet, but decreased in the muscle in response to SL inclusion, although not statistically significant. Overall saturated fatty acids (SFAs) remained constant in the whole body, while they increased in the muscle despite reductions in the diet when *Schizochytrium* was included. MUFA and PUFA levels in whole body and muscle followed levels in the diets.

**Table 4:** Proximate (in % OS) and fatty acid (in % fatty acids) whole‐body composition of Atlantic salmon after 8 weeks of feeding the experimental diets.

**Proximate composition (% OS)**	**CD**	**SL2**	**SL14**	**ANOVA**

Water	69.69 ± 0.22	69.60 ± 0.39	69.19 ± 0.37	ns
Crude protein	17.88 ± 0.12	17.97 ± 0.04	17.95 ± 0.09	ns
Crude lipid	10.48 ± 0.11	10.47 ± 0.47	10.86 ± 0.4	ns
Ash	1.94 ± 0.02	1.95 ± 0.04	2.01 ± 0.05	ns
Energy	8.29 ± 0.07	8.36 ± 0.2	8.51 ± 0.17	ns

**Fatty acid composition (g/100 g FA)**				

C14:0	2.08 ± 0.02	2.06 ± 0.03	2.04 ± 0.01	ns
C16:0	13.00 ± 0.08^a^	13.09 ± 0.21^a^	13.66 ± 0.04^b^	0.023
C18:0	3.04 ± 0.01^a^	3.11 ± 0.08^a^	2.78 ± 0.03^b^	0.008
C20:0	0.35 ± 0^a^	0.34 ± 0^b^	0.31 ± 0^c^	<0.001
Total SFA	19.08 ± 0.08	19.19 ± 0.31	19.39 ± 0.02	ns
C16:1n9	0.35 ± 0^a^	0.32 ± 0.01^a^	0.27 ± 0.01^b^	0.003
C16:1n7	2.16 ± 0.03	2.06 ± 0.05	2.03 ± 0.01	ns
C16:3n4	0.74 ± 0.01^a^	0.73 ± 0.01^a^	0.67 ± 0.01^b^	0.002
C18:1n9	37.19 ± 0.05^a^	36.24 ± 0.04^b^	30.68 ± 0.3^c^	<0.001
C18:1n7	2.6 ± 0^a^	2.54 ± 0.01^b^	2.37 ± 0.02^c^	<0.001
C20:1n11	0.43 ± 0^a^	0.43 ± 0.01^ab^	0.4 ± 0^b^	0.024
C20:1n9	5.19 ± 0.08^a^	5.18 ± 0.1^a^	4.69 ± 0.11^b^	0.017
C22:1n11	3.64 ± 0.02	3.72 ± 0.03	3.56 ± 0.08	ns
C22:1n9	0.47 ± 0^a^	0.46 ± 0^a^	0.42 ± 0^b^	<0.001
C24:1n9	0.44 ± 0	0.43 ± 0.01	0.43 ± 0.01	ns
**Fatty acid composition (g/100 g FA)**	**CD**	**SL2**	**SL14**	**ANOVA**

Total MUFA	52.9 ± 0.11^a^	51.79 ± 0.06^b^	45.26 ± 0.21^c^	<0.001
C18:2n6	11.48 ± 0.12^a^	11.41 ± 0.16^a^	10.2 ± 0.03^b^	<0.001
C18:3n6	0.31 ± 0.02^a^	0.24 ± 0.01^b^	0.18 ± 0.01^b^	0.001
C20:2n6	0.87 ± 0.01^ab^	0.91 ± 0.01^a^	0.82 ± 0.01^b^	0.005
C20:3n6	0.6 ± 0.03^a^	0.51 ± 0.01^b^	0.34 ± 0.01^c^	<0.001
C20:4n6	0.33 ± 0.01^a^	0.31 ± 0.01^a^	0.39 ± 0.01^b^	0.005
C22:5n6	0.13 ± 0^a^	0.32 ± 0^b^	1.38 ± 0.03^c^	<0.001
Total n6‐PUFA	13.85 ± 0.08	13.81 ± 0.17	13.44 ± 0.03	ns
C18:4n3	0.84 ± 0.03^a^	0.75 ± 0.01^b^	0.73 ± 0^b^	0.006
C20:3n3	0.14 ± 0.01	0.13 ± 0.01	0.12 ± 0.01	ns
C20:4n3	0.51 ± 0.01^a^	0.52 ± 0.01^a^	0.56 ± 0.01^b^	0.007
C20:5n3	1.37 ± 0.01^a^	1.38 ± 0.02^a^	1.57 ± 0.04^b^	0.003
C22:5n3	0.72 ± 0^a^	0.72 ± 0.02^a^	0.83 ± 0.03^b^	0.008
C22:6n3	6.06 ± 0.06^a^	7.11 ± 0.1^b^	13.71 ± 0.2^c^	<0.001
Total n3‐PUFA	11.48 ± 0.1^a^	12.54 ± 0.1^b^	19.4 ± 0.25^c^	<0.001
Total PUFA	26.19 ± 0.03^a^	27.2 ± 0.26^b^	33.63 ± 0.22^c^	<0.001
n3‐HUFA	8.93 ± 0.06^a^	10 ± 0.13^b^	16.94 ± 0.27^c^	<0.001

*Note:* Only fatty acids with a concentration exceeding 20 mg/100 g fatty acids are displayed. Data is presented as mean ± SEM as g/100 g FA, with *n* = 3 tanks per treatment. A significant difference (*p*  < 0.05) among diet groups was assessed by Tukey’s multiple comparisons, and values not sharing common letters are significantly different.

**Table 5:** Fatty acid composition of Atlantic salmon muscle fed experimental diets for 8 weeks.

Fatty acid composition (g/100 g FA)	CD	SL2	SL14	ANOVA
C14:0	1.89 ± 0.03	1.88 ± 0.03	1.91 ± 0.04	ns
C16:0	12.76 ± 0.17^a^	13.05 ± 0.14^a^	14.08 ± 0.24^b^	0.006
C18:0	3.08 ± 0.02^a^	2.99 ± 0.03^a^	2.8 ± 0.05^b^	0.003
C20:0	0.33 ± 0^a^	0.33 ± 0^a^	0.3 ± 0.01^b^	0.004
Total SFA	18.69 ± 0.17^a^	18.87 ± 0.15^ab^	19.66 ± 0.26^b^	0.03
C16:1n9	0.31 ± 0.01^a^	0.31 ± 0^a^	0.25 ± 0.01^b^	0.004
C16:1n7	1.98 ± 0.05	1.92 ± 0.04	1.78 ± 0.13	ns
C16:3n4	0.73 ± 0.01	0.75 ± 0.02	0.68 ± 0.01	ns
C18:1n9	35.31 ± 0.47^a^	34.8 ± 0.24^a^	28.51 ± 0.55^b^	<0.001
C18:1n7	2.56 ± 0.03^a^	2.51 ± 0.02^a^	2.24 ± 0.02^b^	<0.001
C20:1n11	0.45 ± 0.01	0.44 ± 0.01	0.4 ± 0.01	0.022
C20:1n9	4.44 ± 0.07^a^	4.44 ± 0.09^a^	3.94 ± 0.11^b^	0.013
C22:1n11	3.4 ± 0.06	3.47 ± 0.08	3.5 ± 0.12	ns
C22:1n9	0.46 ± 0.01^a^	0.46 ± 0.01^a^	0.41 ± 0.01^b^	0.01
C24:1n9	0.43 ± 0.01	0.43 ± 0.01	0.41 ± 0.01	ns
Total MUFA	49.74 ± 0.59^a^	49.17 ± 0.34^a^	41.81 ± 0.89^b^	<0.001
C18:2n6	11.17 ± 0.14^a^	10.97 ± 0.08^a^	9.38 ± 0.23^b^	<0.001
C18:3n6	0.26 ± 0.01^a^	0.23 ± 0.01^a^	0.16 ± 0^b^	<0.001
C20:2n6	0.91 ± 0.02^a^	0.89 ± 0.01^ab^	0.76 ± 0.02^b^	0.008
C20:3n6	0.65 ± 0.03^a^	0.57 ± 0.02^a^	0.34 ± 0.01^b^	<0.001
C20 : 4n6	0.38 ± 0.02	0.37 ± 0.01	0.43 ± 0.03	ns
C22 : 5n6	0.16 ± 0.01^a^	0.38 ± 0.02^b^	1.61 ± 0.06^c^	<0.0001
Total n6‐PUFA	13.65 ± 0.12	13.53 ± 0.08	12.8 ± 0.18	0.021
C18:3n3	2.26 ± 0.02	2.21 ± 0.08	2.04 ± 0.06	ns
C18:4n3	0.76 ± 0.02^a^	0.73 ± 0.01^ab^	0.67 ± 0.01^b^	0.013
C20:4n3	0.55 ± 0.01	0.52 ± 0.01	0.53 ± 0.01	ns
C20:5n3	1.71 ± 0.06	1.65 ± 0.03	1.59 ± 0.04	ns
C22:5n3	0.88 ± 0.02^a^	0.81 ± 0.01^b^	0.83 ± 0.01^ab^	0.044
C22:6n3	8.81 ± 0.52^a^	9.53 ± 0.27^a^	17.06 ± 0.86^b^	<0.001
Total n3‐PUFA	15.31 ± 0.56^a^	15.8 ± 0.34^a^	23.03 ± 0.83^b^	<0.001
Total PUFA	29.81 ± 0.48^a^	30.19 ± 0.36^a^	36.62 ± 0.67^b^	<0.001
n3‐HUFA	12.29 ± 0.59^a^	12.86 ± 0.3^a^	20.31 ± 0.89^b^	<0.001

*Note:* Only fatty acids with a concentration exceeding 20 mg/100 g fatty acids are displayed. Data is presented as mean ± SEM as g/100 g FA, with *n* = 9 individuals per treatment. A significant difference (*p*  < 0.05 ) among diet groups was assessed by Tukey’s multiple comparisons, and values not sharing common letters are significantly different.

### 3.3. Intestinal Microbiota

**Figure 2:**
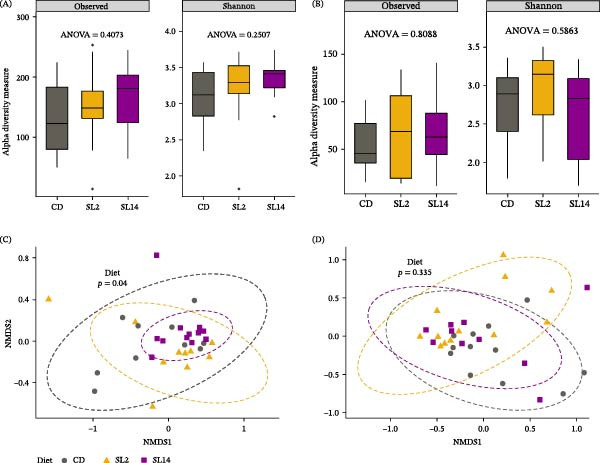
Alpha and beta‐diversity of the digesta and intestinal mucosa of the hindgut of Atlantic salmon fed with *S. limacinum*‐enriched diets for 8 weeks. The fish received either a control diet (CD), a diet containing 2% (SL2), or 14 % (SL14) *S. limacinum*. Observed ASVs and Shannon diversity index of digesta (A) and mucosa (B) do not differ among diets tested by one‐way ANOVA. Nonmetric multidimensional scaling on Bray–Curtis distances based on weighted genus‐level data revealed significant differences between dietary treatments in digesta (C) but not in intestinal mucosa (D) according to PERMANOVA; *n* = 10–11 per diet.

**Figure 3:**
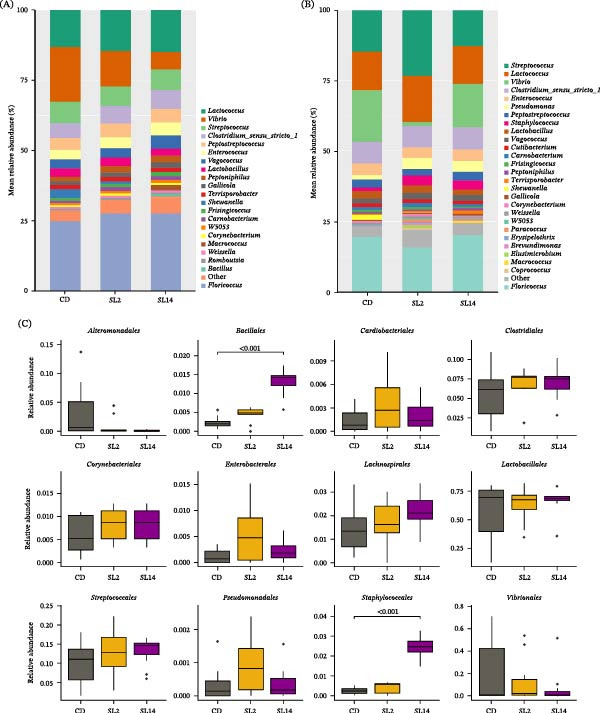
Microbial composition at the genus level of the digesta (A) and intestinal mucosa (B) of Atlantic salmon fed diets enriched with *S. limacinum* for 8 weeks. Relative abundance of bacterial families for the abundant families in digesta (C). The fish received either a control diet (CD), a diet containing 2% (SL2), or 14 % (SL14) *S. limacinum*. Relative abundance of microbiota at the genus level per diet is displayed in a stacked bar plot (A). The category “Other” includes taxonomical clades with an overall abundance of <0.5%.

16S rRNA amplicon sequencing of digesta and mucosa samples revealed a higher alpha‐diversity (observed ASV’s and Shannon Diversity) in digesta than in mucosa samples (Figure 2A,B). Alpha‐diversity in digesta increased slightly with *Schizochytrium* inclusion (Figure 2A), but this proved to be nonsignificant (*p* = 0.41). In the mucosa, alpha‐diversity also remained unaffected by the diet (Figure 2B). Beta diversity in the digesta (Figure 2C), but not in mucosa (Figure 2D), displayed as phylogenetically weighted Bray–Curtis distances using nonmetric multidimensional scaling (NMDS), was significantly modulated by the diet (p = 0.04). Beta diversity of the digesta of fish fed SL14 was significantly different from fish fed CD (*p* = 0.02; Table S2). *Firmicutes* was the most abundant phylum associated with the digesta, followed by *Proteobacteria* (Figure S1). On the genus level, *Floricoccus* was most abundant in digesta and mucosa (Figure 3A,B). In the digesta, it accounted for 26% relative abundance, followed by *Lactococcus* (13%) and *Vibrio* (11%) across treatments (Figure 3A). Bacteria belonging to the families *Bacillales* and *Staphylococcales* were significantly more abundant in the digesta of fish fed SL14 as compared to the control (*p*  < 0.001; Figure 3C). On the genus level, relative abundance of *Shewanella* was reduced in fish fed SL14 (*p* = 0.057), while the genera *Vagococcus* (*p* = 0.052) and *Frisingicoccus* (*p* = 0.055) were both increased, albeit none of the comparisons reached statistical significance. Bacterial communities associated with the mucosa were also dominated by *Firmicutes* (Figure S1), and *Floricoccus* (15%) and Streptococcus (14%) were the two most abundant genera, followed by *Lactococcus* and *Vibrio* (Figure 3B).

To explore potential physiological links between health indicators and bacterial genera in the digesta and mucosa irrespective of the given diet, a Pearson correlation analysis was conducted. Several bacterial genera in digesta and mucosa showed a correlation with plasma and organ health indicators (Figure S2), although none of the correlations remained significant following *p*‐value corrections for multiple correlation testing. Plasma cortisol levels were positively correlated with the relative abundance of *Floricoccus* (*r* = 0.43), *Streptococcus* (*r* = 0.42), *Terrisprobacter* (*r* = 0.37), and *Lactobacillus* (*r* = 0.37) in the digesta (Figure S2A) and *Lactococcus* (*r* = 0.37), *Weissella* (*r* = 0.38), and *Vagococcus* (*r* = 0.45) in the mucosa (Figure S2B). The relative abundance of *Lactobacillus* in the mucosa was furthermore negatively correlated with the spleen‐somatic index (*r* = −0.37).

### 3.4. Response to Acute Stress

**Figure 4:**
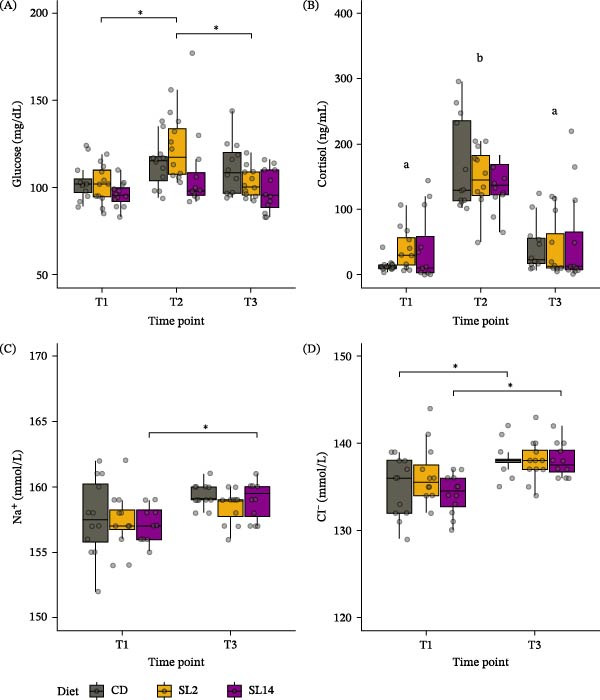
Plasma glucose (A), cortisol (B), sodium (C), and chloride ions (D) of Atlantic salmon fed with *S. limacinum*‐enriched diets for 8 weeks and sampled before (T1), 1 h after (T2), and 18 h after (T3) exposure to peracetic acid‐based disinfectant. The fish received either a control diet (CD), a diet containing 2% (SL2), or 14% (SL14) *S. limacinum*. Data is presented as boxplots with median, *n* = 12. Statistical significance (*p* < 0.05) was assessed by Tukey multiple comparisons, and significant differences within a diet group across time are indicated by  ^∗^, while differences among timepoints are marked by different letters.

Treatment with PAA induced a systemic stress response, which caused an increase in plasma glucose and cortisol levels (Figure 4). No significant differences among diets were detected per timepoint, whereas a significant interaction among diet and timepoint was found for some variables (*p*  < 0.05). Glucose levels increased in fish fed SL2 1 h after exposure to PAA (*p* = 0.005) and decreased again 18 h after treatment (*p* = 0.009), while they remained unchanged in fish fed SL14 and CD (Figure 4A). Cortisol increased strongly in all groups 1 h after PAA treatment (*p* < 0.001) with a subsequent return to initial levels 18 h after treatment (Figure 4B). Sodium ions increased in plasma after exposure to PAA only in fish fed SL14 (*p* = 0.036; Figure 4C), whereas chloride ions increased slightly in fish fed CD (*p* = 0.01) and SL14 (*p* = 0.002; Figure 4D).

## 4. Discussion

Including the heterotrophic microalgae *S. limacinum* in the diets for Atlantic salmon led to significant improvements in growth, caused by the improved feed conversion efficiency in our study. This improvement was seen in fish fed diets containing 14% *Schizochytrium* and, to a lesser degree, in fish fed 2%. Similar to our results, Sarker et al. [47] report improved feed conversion efficiency in Nile tilapia fed with increasing levels of *Schizochytrium*. Improvements in FCR can arise from enhanced digestibility and more efficient metabolic utilization of nutrients. Higher levels of dietary DHA have been shown to improve intestinal health in Atlantic salmon [48] and zebrafish [49], which may partially explain the observed improvement in feed conversion efficiency. We did not detect these improvements in a previous study [28], where Atlantic salmon smolts were fed diets containing 8% *Schizochytrium*. The improved protein retention in fish fed *Schizochytrium* in this study may further indicate higher protein digestibility or more efficient protein utilization. Although previous studies in salmonids showed no effect of *Schizochytrium* on protein digestibility [8, 50], a study in dogs revealed significantly improved protein digestibility when *Schizochytrium* was supplemented to the diet [51]. The observed improvements in protein utilization indicate that Schizochytrium is not only a promising source of DHA but also contains high‐quality protein. Therefore, future studies should particularly investigate the amino acid profile of Schizochytrium when it is included as a whole‐cell product in feed formulations.

The minimal requirement for EPA and DHA to meet nutritional demands in Atlantic salmon is often assumed to be ~0.5% EPA + DHA in the diet [29, 52]. However, life stage and environmental factors can increase the requirement to levels larger than 1% [5, 48, 53]. The present study demonstrates that the provision of excess DHA in the diet of levels that are assumed to have no effect on growth [6, 54] can still improve feed conversion and thus growth. The intensive RAS rearing environment may have increased the salmon´s PUFA demand due to stress‐induced ß‐oxidation [55, 56]. Consequently, improvements in FCR were detected even at high dietary DHA concentrations in this experiment. Nevertheless, we cannot fully rule out that other compounds present in *Schizochytrium* may have been responsible for the observed improvement in the feed conversion efficiency. The lack of any effect on the fish’s condition and organ indices is in line with previous findings [9, 57] and confirms the overall good nutritional status of the fish.

Fatty acids and their ratios in the diet generally are also reflected in the fatty acid composition of the fish [54, 58, 59]. Different responses to dietary fatty acid ratios can often be seen in the fatty acid profile of neutral and polar lipids [60]. Deviations can indicate alterations in the fatty acid synthesis as well as degradation in the form of ß‐oxidation. PUFAs are highly digestible for salmonids [58], and high contents of DHA in whole‐body and muscle samples of the salmon reflected uptake of DHA from *Schizochytrium*, without the need to disrupt cells or extract oil [9, 28]. Nevertheless, uptake efficiency is reduced at high dietary DHA concentrations, as found in the present and previous studies [53]. Therefore, dietary DHA levels should match the actual requirements for efficient utilization. In the current study, in addition to our previous research [28], *Schizochytrium* inclusion reduced the concentration of DGLA in both the muscle and the whole body of the salmon. DGLA is synthesized from linoleic acid via gamma‐linolenic acid and can be further converted to arachidonic acid (ARA) or 1‐series prostaglandins [61]. Increased concentrations of ARA‐derived metabolites may contribute to an exaggerated inflammatory response [62, 63]. The linolenic acid content decreased with *Schizochytrium* inclusion, likely resulting in reduced biosynthesis of DGLA.

Overall, the fatty acid profile was rather similar between whole‐body and muscle samples, but EPA in muscle samples did not increase in response to the slightly increased levels in the SL14 diet. This may indicate increased conversion to eicosanoids or increased beta‐oxidation in the muscle [54]. Furthermore, SFA levels in the muscle varied in relation to the given diet, whereas they remained constant in the whole body despite decreasing levels in the diets. *Schizochytrium* contains significant amounts of palmitic acid (C16:0; [8, 9]), and to balance the diet formulation, palm oil content was reduced with increasing inclusion of *Schizochytrium* in the diet, resulting in lower overall contents of C16:0 in the diets. A tight and complex regulation of C16:0 contents on the tissue level [64] may explain that levels of palmitic acid in both muscle and whole‐body samples of salmon fed *Schizochytrium* remained constant or increased, despite decreasing levels in the diet. This may indicate potential overcompensation via de novo synthesis.

Given that we observed clear improvements in growth performance when *Schizochytrium* was included in the diet, this tempted us to explore the effects of this microalgae on the intestinal microbiota, an important mediator of host health and performance [65]. Our findings indicate that including *Schizochytrium* in the diet changed beta‐diversity and slightly increased alpha‐diversity in the digesta of Atlantic salmon, while these remained unaltered in the mucosa. This seems to agree with the current understanding that resident bacteria within the intestinal mucosa of fish are predominantly controlled via host‐related factors, while transient bacteria associated with the digesta appear to be more affected by environmental and dietary influences [66, 67]. Previous studies on rainbow trout [68] and Nile tilapia [69, 70] did not detect any effects on beta‐diversity when feeding *Schizochytrium*‐enriched diets. However, de Souza et al. [69] found increased Chao‐1 diversity and a trend of increased Shannon diversity in tilapia fed with 1.2% *Schizochytrium*. The present study revealed high levels of alpha‐diversity in the digesta and mucosa. This finding contrasts the results of previous studies in Atlantic salmon, which indicated a comparatively simple bacterial community in the digesta and intestinal mucosa dominated by very few species [71]. An important difference of this study compared to most of the previous studies is that the fish were raised in a RAS. RAS have been shown to harbor a rich source of diverse bacteria, and the diversity of the rearing water in RAS is often much higher than that of flow‐through systems [72, 73]. This generally higher diversity, as well as specific water quality characteristics such as dissolved metabolites and particles, could give rise to a more diverse intestinal microbiome found in RAS‐reared fish (this study) compared to fish held in flow‐through systems. In fact, Atlantic salmon juveniles reared in RAS also showed twice the number of observed ASVs compared to juveniles in flow‐through systems [74].

Most bacterial taxa found in the present study were classified as *Firmicutes*, *Proteobacteria*, and *Actinobacteria* in both digesta and intestinal mucosa, which agrees with findings in fish [75] and more specifically Atlantic salmon [76]. Our comparisons at the genus level did not reveal any statistically significant differences in relative abundance, as variability among individuals was generally large. However, there was a trend that the bacteria *Vagococcus* and *Frisingicoccus*, belonging to the phylum *Firmicutes*, increased in abundance in the digesta of fish fed SL14. Feeding Nile tilapia diets supplemented with 1.2% *Schizochytrium* also increased the relative abundance of *Firmicutes* and, more specifically, bacteria of the class *Clostridia* [69]. A study where rainbow trout were fed a diet enriched with 5% *Schizochytrium* for 15 weeks found that the relative abundance of *Streptococcus*, *Leuconostoc*, and *Weissella* was associated with fish fed the diet with *Schizochytrium* [68], indicating that *Schizochytrium* inclusion in diets for fish may specifically promote bacteria of the phyla *Firmicutes*. This is potentially linked to the presence of complex carbohydrates (fibers) in the cell wall of Schizochytrium, promoting the growth of bacteria able to use these as metabolic fuel. Within Firmicutes, many lactic acid bacteria are considered promising probiotics for aquaculture, and several species have shown to produce antibacterial substances active against fish pathogens [77].

Beyond dietary influences, host‐associated factors play an important role in influencing the composition of the intestinal microbiota. Correlating host‐associated factors with the abundance of specific bacterial genera of the intestinal microbiome can give first indications about potential connections. Results of this study suggest a connection between the primary stress hormone cortisol and the abundance of Lactobacillales in digesta and mucosa. A previous study in Atlantic salmon also revealed specific associations between bacterial taxa in the digesta and fecal cortisol levels [78]. However, manipulative studies are needed to fully understand the role the stress hormone cortisol has on the intestinal microbiota of fish.

Treatment with PAA induced an acute stress response in the present study, which is in line with previous findings in Atlantic salmon [21, 79] and rainbow trout [80, 81]. While the dynamics of the cortisol response were not influenced by the diet [30] and followed those classically observed for an acute stress response [82, 83], glucose concentrations in the plasma increased only in fish fed 2% *Schizochytrium*. This contrasts an earlier study in zebrafish, which found that a diet enriched with 12% *Schizochytrum* sp. resulted in increased glucose levels in the humoral fluid [84]. The overall link between feeding Schizochytrium and increased plasma glucose levels remains unclear, and future studies should investigate specific compounds present in Schizchytrium that could interact with the glucose response. Acute stress increases the gill ventilation rate and branchial blood flow, resulting in increased blood perfusion in the gills [85]. This increased permeability of the gill surface in a hyperosmotic environment results in an elevated influx of ions affecting plasma osmolality, also known as the osmorespiratory compromise. Plasma ions were slightly elevated 18 h after PAA treatment but remained controlled within physiological limits. Taken together, these results confirm that acute PAA treatment is a mild stressor for Atlantic salmon, while feeding different levels of the DHA‐rich microalgae *Schizochytrium* only marginally affected the glucose and ion regulatory response to stress.

## 5. Conclusion

Future reductions of marine ingredients in fish feed are the fundamental requirement for expanding aquaculture production. At the same time, aquafeeds need to promote the health of fish and support them in overcoming challenges associated with increasingly intensive production. This is particularly relevant with the expansion of RAS, for which optimal requirements of certain functional compounds in the feed are unknown even for highly studied species such as Atlantic salmon. Enriching feeds with a sustainable source of DHA using the microalgae *Schizochytrium* can improve the growth performance and stress resilience of Atlantic salmon farmed in RAS. Improvements in performance and health status were already detectable at 2% inclusion of *Schizochytrium*, while intestinal microbiota remained rather unaffected by inclusion of the microalgae, indicating a limited role to modulate the intestinal microbiota. Future studies should particularly focus on the interaction of stress with high levels of PUFAs from both a mechanistic and applied perspective, utilizing laboratory experiments as well as field trials under challenging conditions.

## Author Contributions

Jonas Mueller designed the experiment with input from Carsten Schulz, Henrike Seibel, and Anna Simon. Jonas Mueller and Jannick Ehlers performed the experiment, conducted the laboratory work, and acquired the data. Joachim Molkentin and Irene Lautenschläger conducted fatty acid analysis, while Stéphanie C. Hornburg and Corinna Bang took care of the microbiota extraction and sequencing. Jonas Mueller and Marvin Suhr analyzed the data, performed statistical evaluation, and prepared figures. Jonas Mueller wrote the manuscript.

## Funding

This work was funded by the BMBF within the project BioFiA (Project Number 031B0915). Microbiota sequencing received infrastructure support from the DFG Excellence Cluster 2167 “Precision Medicine in Chronic Inflammation” (PMI) and the DFG Research Unit 5042 “miTarget”. Jonas Mueller was supported by a scholarship from the H. Wilhelm Schaumann Stiftung. Open Access funding enabled and organized by Projekt DEAL.

## Disclosure

All authors read and approved the final version of the manuscript.

## Conflicts of Interest

The authors declare no conflicts of interest.

## Supporting Information

Additional supporting information can be found online in the Supporting Information section.

## Supporting information


**Supporting Information** The supporting material contains the following tables and figures: Table S1 ANOVA and Tukey’s multiple comparison results for the effect of diet on the production‐related parameters and organ indices. Table S2 PERMANOVA results for the effect of diet on the intestinal digesta and mucus microbiota beta‐diversity as well as pairwise comparisons using permutation MANOVAs on a distance matrix. Figure S1 Relative abundance of phyla in the digesta (A) and mucosa (B) of Atlantic salmon fed either a control diet (CD), a diet containing 2% (SL2), or 14% (SL14) *S. limacinuum*. Figure S2 Correlation matrix heatmap for bacterial genera in digesta (A) and mucosa (B) with plasma and biometric health indicators in Atlantic salmon.

## Data Availability

The sequencing data that support the findings of this study are openly available in the Sequence Read Archive at https://www.ncbi.nlm.nih.gov/sra/, reference number PRJNA1083322. Other data is available from the corresponding author upon reasonable request.
