# Supplementary material for: DHA‐Rich Algae Feed Modulates Atlantic Salmon Health, Microbiota and Stress Response
Source: Aquac Nutr. 2026 Jul 2;2026:4185490. doi: 10.1155/anu/4185490 (PMC13324814; doi:10.1155/anu/4185490)
Supplement: Supplementary file 1 — Supporting Information The supporting material contains the following tables and figures: Table S1 ANOVA and Tukey’s multiple comparison results for the effect of diet on the production‐related parameters and organ indices. Table S2 PERMANOVA results for the effect of diet on the intestinal digesta and mucus microbiota beta‐diversity as well as pairwise comparisons using permutation MANOVAs on a distance matrix. Figure S1 Relative abundance of phyla in the digesta (A) and mucosa (B) of Atlantic salmon fed either a control diet (CD), a diet containing 2% (SL2), or 14% (SL14) S. limacinuum. Figure S2 Correlation matrix heatmap for bacterial genera in digesta (A) and mucosa (B) with plasma and biometric health indicators in Atlantic salmon. [file ANU-2026-4185490-s001.docx]

Supplementary Material

**DHA-rich algae feed modulates Atlantic salmon health, microbiota and stress response**

Jonas Mueller^1,2^, Marvin Suhr^3^, Joachim Molkentin^4^, Irene Lautenschläger^4^, Jannick Ehlers^2^, Anna Simon^1,2^, Stéphanie C. Hornburg^3^, Corinna Bang^5^, Henrike Seibel^2^, Carsten Schulz^1,2^

*^1^Institute of Animal Breeding and Husbandry, Department for Marine Aquaculture, Kiel University, Kiel, Germany*

*^2^Fraunhofer Research Institution for Individualized and Cell-Based Medical Engineering IMTE, Aquaculture and Aquatic Resources, Büsum, Germany*

*^3^Institute of Animal Nutrition and Physiology, Kiel University, Kiel, Germany*

*^4^Max Rubner-Institute, Department of Safety and Quality of Milk and Fish Products, Kiel, Germany*

*^5^Institute of Clinical Molecular Biology,* *Kiel University, Kiel, Germany*

Table S1 ANOVA and Tukey´s multiple comparison results for the effect of diet on the production related parameters and organ indices

| Variable | ANOVA F | Anova p-value | CD-SL2 | CD-SL14 | SL2-SL14 |
| --- | --- | --- | --- | --- | --- |
| IBW | 0.4 | 0.687 | - | - | - |
| FBW | 9.848 | 0.013 | 0.1605 | 0.0105 | 0.1331 |
| SGR | 31.363 | <0.001 | 0.0924 | 0.0008 | 0.0044 |
| TGC | 27.707 | <0.001 | 0.0636 | 0.0007 | 0.01 |
| FCR | 22.542 | 0.002 | 0.1465 | 0.0014 | 0.0111 |
| PER | 30.856 | <0.001 | 0.1121 | 0.0005 | 0.0044 |
| PRE | 42.172 | <0.001 | 0.0352 | 0.0002 | 0.0028 |
| CF | 2.516 | 0.161 | - | - | - |
| HSI | 0.6448 | 0.558 | - | - | - |
| SSI | 0.158 | 0.8573 | - | - | - |

Table S2 PERMANOVA results for the effect of diet on the intestinal digesta and mucus microbiota beta diversity as well pairwise comparisons using permutation MANOVAs on a distance matrix.

|  | PERMANOVA | | | Pairwise comparisons (p- value) | | |  |
| --- | --- | --- | --- | --- | --- | --- | --- |
|  | **F** | **R^2^** | **Pr(>F)** | **CD - SL2** | **CD - SL14** | **Sl2- SL14** | |
| Digesta | 1.86 | 0.11359 | 0.04 | 0.38 | 0.018 | 0.063 | |
| Mucosa | 1.08 | 0.06534 | 0.335 | 0.22 | 0.44 | 0.4 | |

**B**

**A**


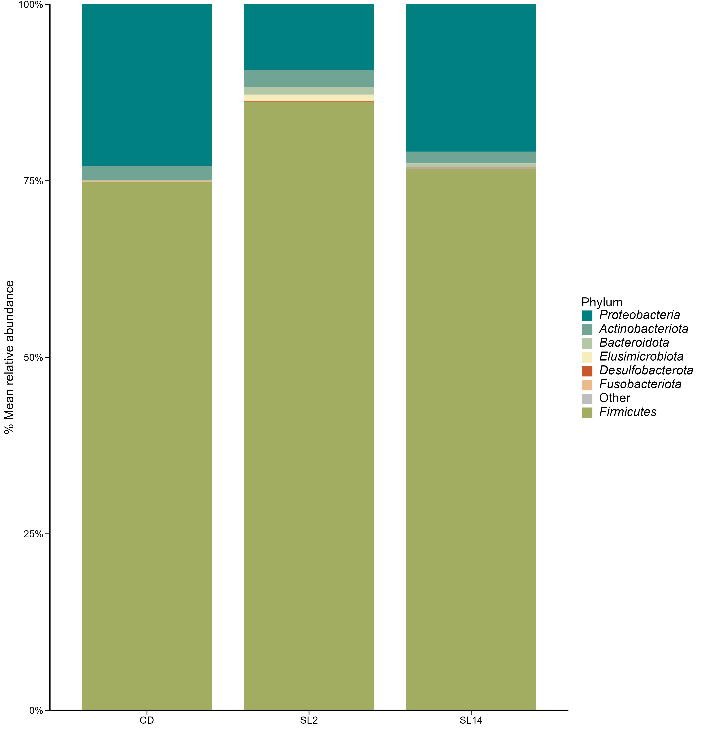


**B**

**A**


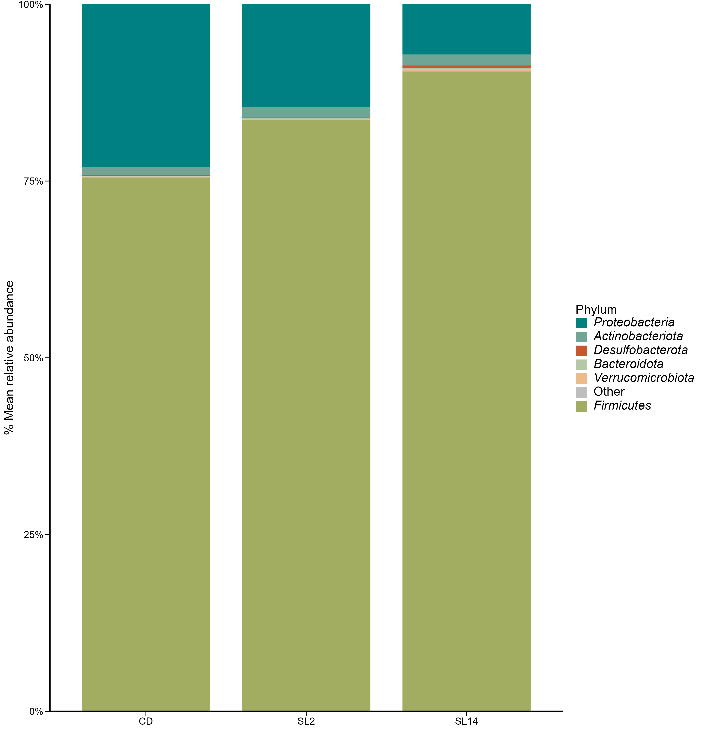


Figure S1 Relative abundance of phyla in the digesta (A) and mucosa (B) of Atlantic salmon fed either a control diet (CD), a diet containing 2% (SL2), or 14 % (SL14) *S. limacinuum*.

**B**

**A**


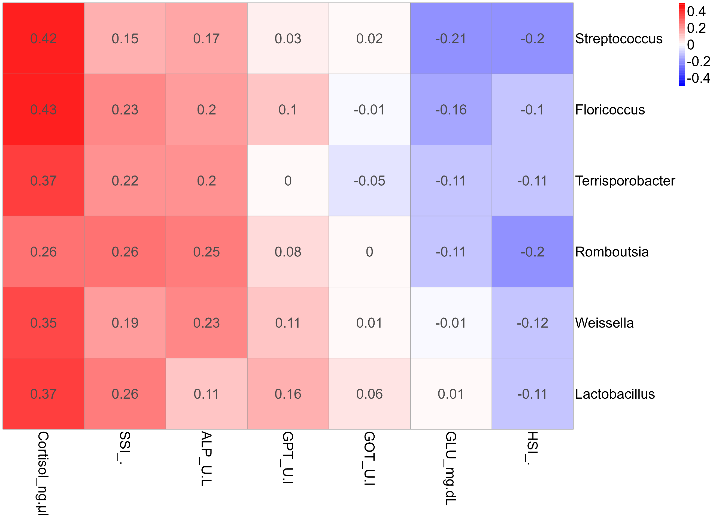

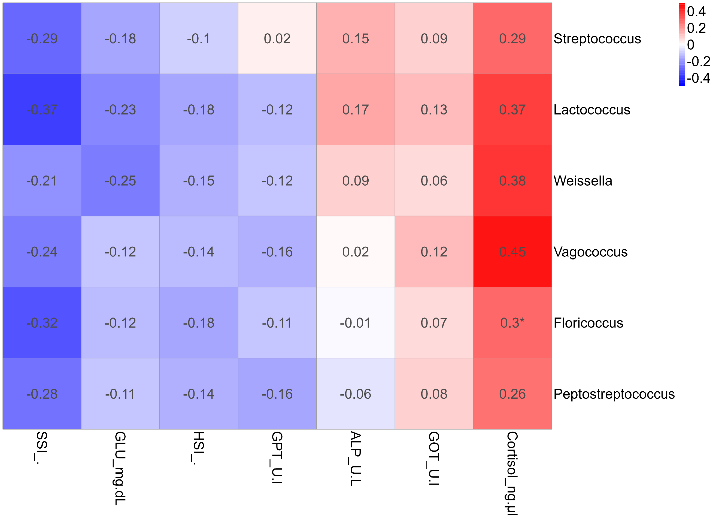


Figure S2 Correlation matrix heatmap for bacterial genera in digesta (A) and mucosa (B) with plasma and biometric health indicators in Atlantic salmon. The heatmap visualizes the correlation coefficients between different bacterial genera (rows) and physiological/biochemical parameters (columns). The color scale represents the strength and direction of the correlations, with red indicating positive correlations and blue indicating negative correlations. The intensity of the color corresponds to the correlation strength, as indicated by the color bar on the right.
